# A case of acinar cell carcinoma originating from the accessory papilla of the duodenum

**DOI:** 10.1186/s40792-024-01872-3

**Published:** 2024-04-16

**Authors:** Kiyoshi Narita, Masataka Okuno, Seiji Natsume, Tomonari Asano, Hisafumi Saito, Masashi Negita, Seiji Ito, Koji Komori, Tetsuya Abe, Kazuo Hara, Nozomi Okuno, Waki Hosoda, Yasuhiro Shimizu

**Affiliations:** 1 Department of Gastroenterological Surgery, Aichi Cancer Center Hospital, 1-1 Kanokoden, Chikusa, Nagoya, Aichi 464-8681 Japan; 2 Department of Gastroenterology, Aichi Cancer Center Hospital, 1-1 Kanokoden, Chikusa, Nagoya, Aichi 464-8681 Japan; 3 Department of Pathology and Molecular Diagnostics, Aichi Cancer Center Hospital, 1-1 Kanokoden, Chikusa, Nagoya, Aichi 464-8681 Japan

**Keywords:** Acinar cell carcinoma, Accessory papilla, Minor papilla, Ectopic pancreas, BCL10

## Abstract

**Case presentation:**

A 61-year-old female was referred to our hospital with a neoplastic lesion in the duodenum. Computed tomography with contrast enhancement revealed a 10-mm tumor in the duodenum. Upper gastrointestinal endoscopy revealed a submucosal tumor-like lesion in the descending part of the duodenum. Endoscopic ultrasound revealed a well-defined hypoechoic tumor. Biopsy and immunohistochemical findings including negative Synaptophysin and Chromogranin A staining and positive Trypsin and BCL10 staining suggested a carcinoma with acinar cell differentiation. Pancreatoduodenectomy was performed, and the resected specimen had a 15-mm solid nodule in the submucosal layer of the duodenum. Pancreatogram of the resected specimen revealed a tumor localized in the accessory papilla region. In histopathological examination, the tumor was found in the submucosa of the duodenum with pancreatic tissue present nearby, and these were separated from the pancreatic parenchyma by the duodenal muscle layer. These findings led to a diagnosis of acinar cell carcinoma originating from the accessory papilla of the duodenum.

**Conclusion:**

Acinar cell carcinoma originating from the accessory papilla of the duodenum is exceptionally rare, with no reported cases to date. The origin was considered to be pancreatic tissue located in the accessory papilla region.

## Background

Acinar cell carcinoma (ACC) is an uncommon neoplasm of the pancreas that shows evidence of acinar differentiation. ACC accounts for roughly 0.5% of pancreatic tumors [1], and ACC outside the pancreas is even more rare. Here we report an extremely rare case of ACC originating from the accessory (minor) papilla of the duodenum, which was revealed by diagnostic imaging and histology.

## Case presentation

**Fig. 1:**
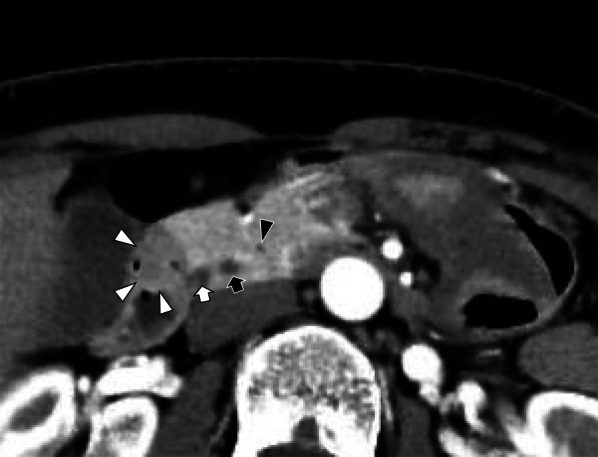
Abdominal contrast-enhanced CT scan. A well-defined 10-mm tumor was observed in the descending part of the duodenum at the border with the pancreas (white triangles). There was no dilatation of the main pancreatic duct (black arrow) and common bile duct (white arrow). The accessory pancreatic duct was identified (black triangle)

**Fig. 2:**
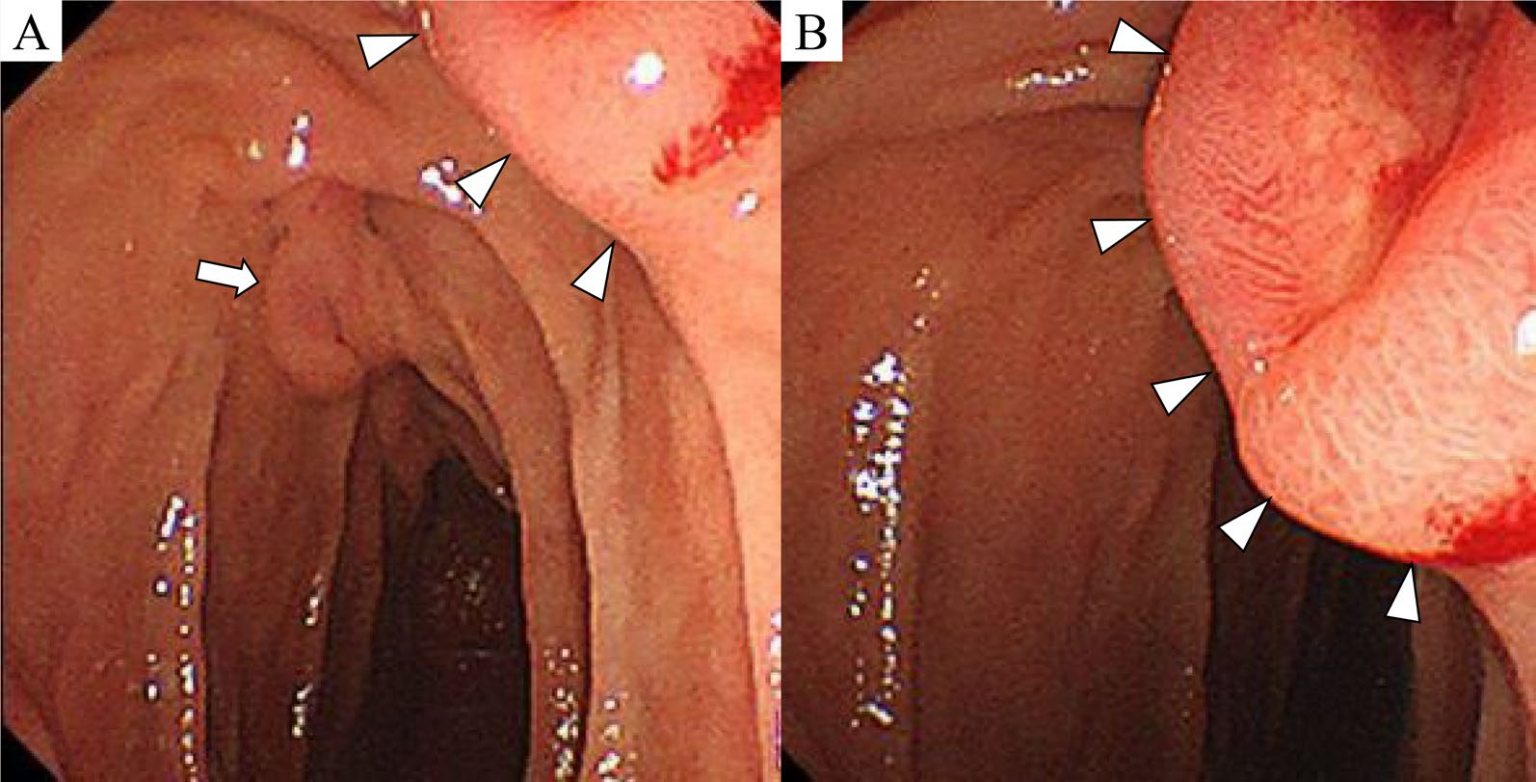
Upper gastrointestinal endoscopy. **A** The tumor (white triangles) was located on the slightly oral side of Vater’s papilla (white arrow). **B** A submucosal tumor-like lesion with a central concavity was observed in the descending part of the duodenum

**Fig. 3:**
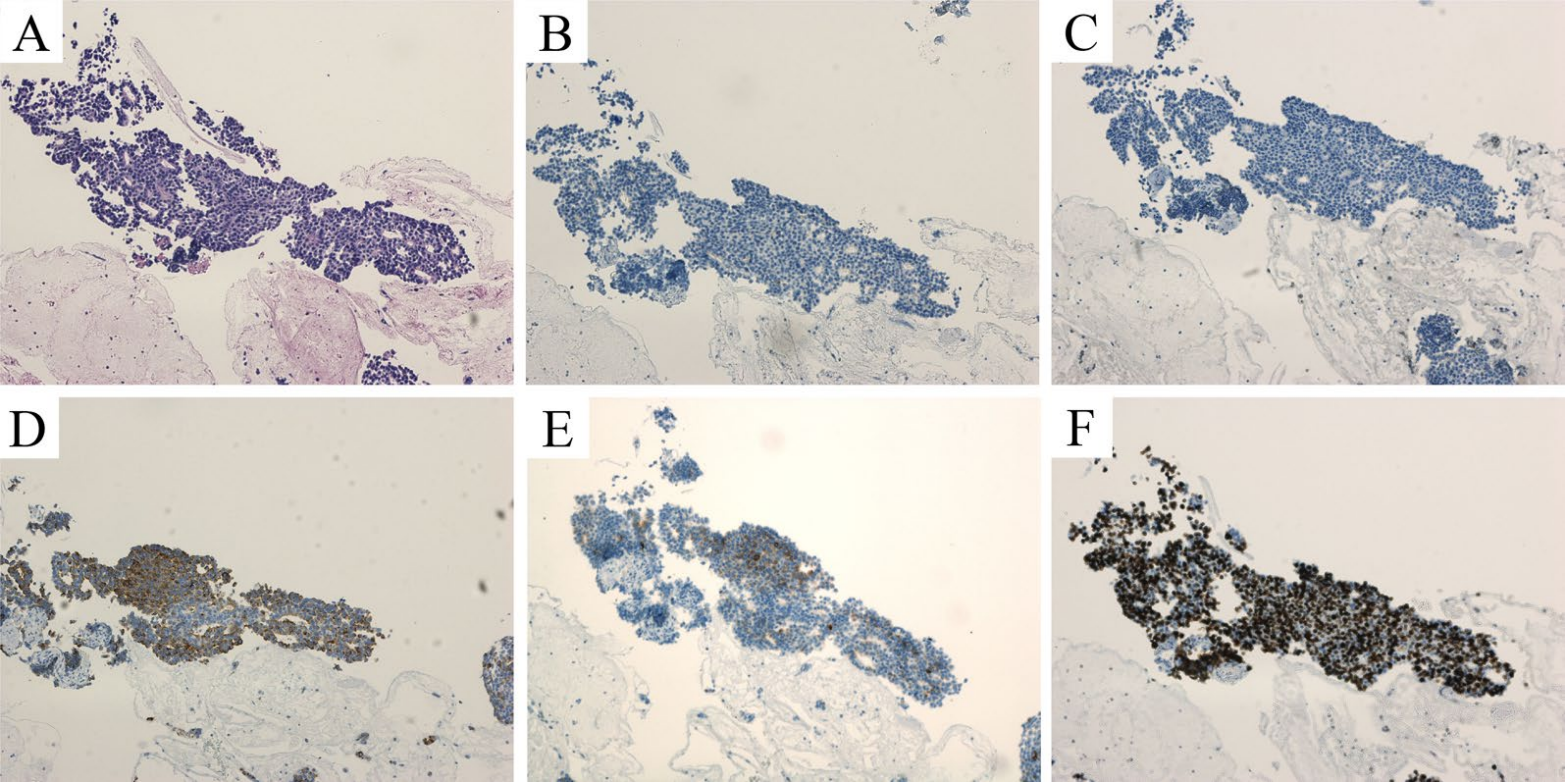
Histopathological and immunohistochemical examinations of biopsies. **A** Hematoxylin and eosin stain (× 10). Neoplastic cells exhibited round nuclei and granular cytoplasm, with focal small gland-like structures. **B** Synaptophysin negative, **C** Chromogranin A negative, **D** Trypsin positive, **E** BCL10 focal positive, **F** Ki-67 index 80%

A 61-year-old female was prescribed iron supplements for iron-deficiency anemia by her family doctor, as progression of anemia had been noted. Upper gastrointestinal endoscopy revealed a neoplastic lesion in the duodenum, and she was referred to our hospital. Her previous upper gastrointestinal endoscopy performed 2 years earlier had shown no abnormalities. Her height was 158.0 cm, and body weight was 51.4 kg. Laboratory findings included a hemoglobin level of 11.7 g/dL, indicating mild anemia, with no significant abnormalities in liver and biliary enzymes, pancreatic enzymes, and tumor markers. Abdominal contrast-enhanced computed tomography (CT) scans revealed a 10-mm tumor protruding into the lumen of the duodenum (Fig. 1). The contrast effect was weaker than the pancreatic parenchyma, and the tumor had a distinct boundary with the pancreas. There was no dilatation of the main pancreatic duct and common bile duct. The accessory pancreatic duct could be recognized. There were no findings of lymph node metastasis or distant metastasis. Upper gastrointestinal endoscopy performed in our hospital revealed a submucosal tumor-like lesion with a central concavity in the descending part of the duodenum on the slightly oral side of Vater’s (major) papilla (Fig. 2). Endoscopic ultrasound (EUS) revealed a well-defined hypoechoic tumor measuring 10 mm. The tumor was in proximity to the pancreatic parenchyma, but we could not confirm the boundary with the pancreatic parenchyma on EUS. Histopathological examination of biopsies obtained by endoscopy and EUS-guided method revealed a solid tumor consisting of neoplastic cells with round nuclei and granular cytoplasm, with focal small gland-like structures (Fig. 3). Immunohistochemical findings included negative Synaptophysin and Chromogranin A staining and positive Trypsin and BCL10 staining. The Ki-67 index was 80%. A diagnosis of carcinoma with acinar cell differentiation was made.

We diagnosed the tumor as duodenal carcinoma cT1b/cN0/cM0 cStage I according to the 8th edition UICC-TNM classification. The tumor appeared to be localized in the accessory papilla region. However, the reason for the expression of specific markers of pancreatic acinar cells remained unclear because the CT scan suggested that the tumor was separated from the pancreatic parenchyma where ACC originally develops.

We performed pancreatoduodenectomy. Intraoperatively, a 10-mm mass without serosal invasion was palpated in the descending part of the duodenum. There were no liver or peritoneal metastases. Intraoperative ultrasound revealed an expanded pancreatic duct in the pancreatic head, which was suspected to be the accessory pancreatic duct. The bile duct was transected at the common bile duct, and the pancreas was divided on the ventral side of the superior mesenteric vein. The resection margins did not reveal tumor cells in rapid pathological examinations. The reconstruction was performed by Child’s procedure. The operative time was 7 h and 1 min, with a blood loss of 190 mL.

**Fig. 4:**
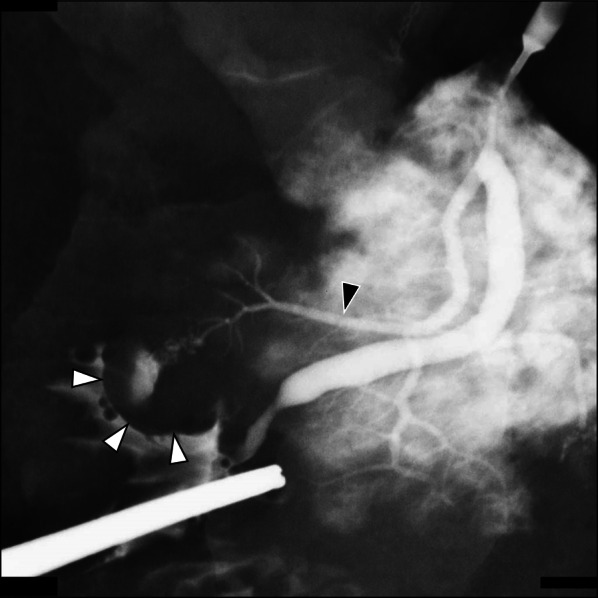
Pancreatogram of the resected specimen. The tumor (white triangles) located at the orifice of the mildly dilated accessory pancreatic duct (black triangle)

**Fig. 5:**
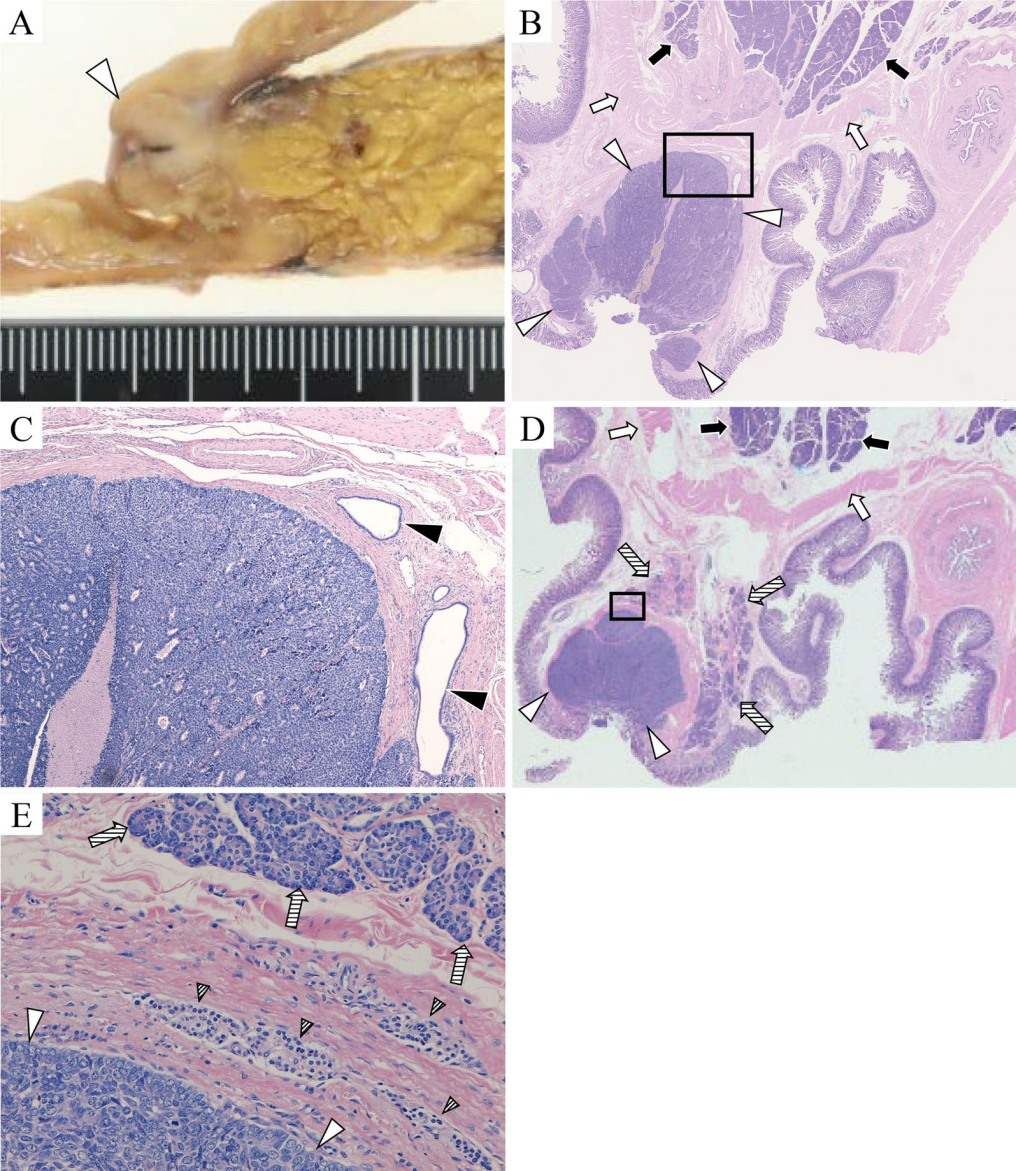
Histopathological examination. **A** Macroscopic findings. The resected specimen revealed a 15-mm white nodule within the submucosa of the duodenum (white triangle). **B** Hematoxylin and eosin stain. The tumor (white triangles) was limited to the submucosa of the duodenum and separated from the pancreatic tissue (black arrows) by the duodenal muscularis (white arrows). **C** Hematoxylin and eosin stain which the enlargement of the black square in **B**. Tumor cells were not detected within the pancreatic duct around the tumor, and the duct was considered the accessory pancreatic duct (black triangles). **D** Hematoxylin and eosin stain at the adjacent section. Normal pancreatic tissue (striped arrows) was observed in the accessory papilla region of the duodenum, which sat in a semicircle around the tumor (white triangles). This normal pancreatic tissue was separated from the pancreatic parenchyma (black arrows) by the duodenal muscle layer (white arrows) and thus considered as pancreatic tissue in the accessory papilla region. **E** Hematoxylin and eosin stain which the enlargement of the black square in **D**. At high magnification, acinar cells (striped arrows) and islets of Langerhans (striped triangles) were seen next to the tumor (white triangles)

The resected specimen had a 15-mm nodule on the oral side of Vater’s papilla. The dilated accessory pancreatic duct was connected to the tumor, as revealed by the pancreatogram of the resected specimen, leading to a diagnosis of a tumor located in the accessory papilla region (Fig. 4). In histopathological examination, we observed the tumor cells growing monotonously in a solid fashion, focally showing gland-like structures (Fig. 5). Immunohistochemically, the tumor was positive for pancreatic acinar markers of Trypsin and BCL10. The tumor was limited to the submucosa of the duodenum, with no continuity with the pancreatic tissue. Beneath the lesion was the accessory pancreatic duct; no tumor cells were identified within the pancreatic ducts around the tumor. We also observed normal pancreatic tissue in the accessory papilla region (Fig. 5D). Notably, the normal pancreatic tissue was located next to the tumor (Fig. 5E), which allowed us to consider the origin of ACC occurring in this rare location. Based on these findings, we diagnosed this tumor as ACC in the accessory papilla region (tumor size 15 × 10 mm, pT1b/pN0/cM0 pStage I). The postoperative course was uneventful, and the patient was discharged on day 15 after surgery. Adjuvant chemotherapy was not administered. The patient has undergone 6 months of postoperative follow-up without any recurrence.

## Discussion

Pancreatic ACC is defined as a tumor that exhibits differentiation toward pancreatic acinar cells [2]. It shows an expansile growth pattern and is primarily found in the pancreatic head [3]. On CT and magnetic resonance imaging, well-defined borders with an outward protrusion are observed. Smaller lesions often exhibit a relatively uniform enhancement pattern, although less intense than that of normal pancreatic parenchyma [4]. Large ACCs can exhibit hemorrhage and cystic changes. Histologically, ACC is characterized by cells with round nuclei, distinct nucleoli, and granular cytoplasm that exhibit acinar-like structures [5].

Differential diagnoses are neuroendocrine carcinoma and solid pseudopapillary neoplasm, and immunohistochemistry is essential for their distinction. In addition to traditional markers such as Trypsin and Lipase, BCL10 has recently been reported to be a useful marker for the diagnosis of ACC [6]. BCL10 is exclusively expressed in normal acini and shows positive expression in 82% of resected ACC cases and 50% of adenosquamous carcinoma cases, while exhibiting negative expression in other subtypes of pancreas neoplasms [6]. In the present case, BCL10 positivity in immunohistochemistry played a key role in the diagnosis of ACC.

The accessory papilla, situated approximately 2 cm proximal to Vater’s papilla on the anterior wall of the descending duodenum, is present in most people [7]. It is often described as a potential secondary drainage route for pancreatic juice and a safety valve to prevent acute pancreatitis, although many aspects of its function remain unclear [7]. The accessory papilla is considered to be formed by the accessory pancreatic duct after penetrating the duodenal muscle layer and the surrounding fibrous connective tissue. However, the accessory papilla is not always surrounded by the sphincter like Vater’s papilla, which is surrounded by the sphincter of Oddi, and thus the border of the accessory papilla is not well-defined [7–9]. Therefore, we refer to this area as “the accessory papilla region” in this report. Pancreatic tissue is known to exist within the accessory papilla region, with a reported frequency of 59.0–76.4% in autopsy cases; this incidence is higher than the 9.2% for Vater’s papilla [10, 11]. Neoplasms in the accessory papilla region rarely occur, except for duodenal adenomas. In our search in PubMed between 1985 and 2023, we found a few reports of neuroendocrine tumor and adenocarcinoma but no reports of ACC.

An intriguing aspect of our case is the tumor’s origin. The most likely origin of ACC occurring in this region is infiltration from pancreatic ACC into the accessory papilla or progression of pancreatic ACC through the pancreatic duct to the accessory papilla. Indeed, ACC reportedly can grow and spread in the pancreatic duct, mimicking pancreatic intraductal neoplasm [2]. However, in the present case, pathological analysis revealed the tumor and the pancreatic parenchyma to be separated by the duodenal muscle layer with no continuity between them. In addition, no tumor cells were found within the surrounding pancreatic duct. Therefore, the scenario of pancreatic ACC extending to the accessory pancreatic duct was denied. Moreover, based on the observation that pancreatic acinar tissue was identified in the accessory papilla region and that it was quite close to the tumor, we think that the tumor may have arisen from this pancreatic tissue found in the accessory papilla region.

There remains a question as to whether the pancreatic tissue in the accessory papilla region could be considered an ectopic pancreas. The ectopic pancreas is defined as pancreatic tissue that lacks continuity with the pancreatic parenchyma and differs in vascular supply [12]. It is commonly found in the gastrointestinal tract, such as the duodenum, stomach, and jejunum [13]. Although there may be controversy as to whether the vascular supply of the accessory papilla and the pancreas is the same or different, pancreatic tissue in the accessory papilla region, as observed in the present case, lacks continuity with the pancreatic parenchyma. When considering the development of the pancreas in the fetus, the accessory papilla arises from the dorsal pancreatic bud of the duodenum, and during its formation acinar cell differentiation may occur in the region where it eventually becomes an accessory papilla in adulthood. Indeed, pancreatic tissue was observed in the accessory papilla in 59.0–76.4% of autopsy cases [10, 11]. Therefore, it may be possible to consider the pancreatic tissue in the accessory papilla to be an ectopic pancreas, although its formation is likely linked to the development of the pancreas. We conducted a literature review of ACC originating from ectopic pancreatic tissue. Although the occurrence of this condition is extremely rare, our searches in PubMed between 1999 and 2023 identified 15 case reports, including 8 in the stomach, 2 in the jejunum, 1 in the liver, and 4 in the duodenum; 2 of the duodenal cases involved the duodenal wall and the remaining 2 involved Vater’s papilla [14–28]. This case presents the first report of ACC of the accessory papilla.

The primary treatment for resectable pancreatic ACC is surgery, and the prognosis is typically better compared to pancreatic ductal adenocarcinoma [29]. While there is no standard regimen for both adjuvant chemotherapy and chemotherapy for recurrent or advanced cases of ACC [30], it is often conducted following a regimen similar to that used for pancreatic ductal adenocarcinoma [30]. The most common forms of recurrence in pancreatic ACC are liver metastasis followed by peritoneal metastasis [29]. Our case was diagnosed and treated at a relatively early stage, and a favorable prognosis, similar to that with pancreatic ACC, could be expected. However, given that a few reports exist on cases of ACC originating from ectopic pancreatic tissue, and as its prognosis still remains unclear, close follow-up is essential.

## Conclusions

We experienced an extremely rare case of ACC originating from the accessory papilla of the duodenum. The tumor appeared to have arisen from pancreatic tissue in the accessory papilla region, which is intriguing given the histological structure of the accessory papilla.

## Data Availability

Data sharing is not applicable since no datasets were generated or analyzed during the present study.

## Abbreviations

ACC: Acinar cell carcinoma
CT: Computed tomography
EUS: Endoscopic ultrasound
